# Hydrothermal Synthesis and Acetylene Sensing Properties of Variety Low Dimensional Zinc Oxide Nanostructures

**DOI:** 10.1155/2014/489170

**Published:** 2014-01-30

**Authors:** Qu Zhou, Weigen Chen, Shudi Peng, Wen Zeng

**Affiliations:** ^1^State Key Laboratory of Power Transmission Equipment & System Security and New Technology, Chongqing University, Chongqing 400030, China; ^2^Chongqing Electric Power Research Institute, Chongqing 401123, China; ^3^College of Materials Science and Engineering, Chongqing University, Chongqing 400030, China

## Abstract

Various morphologies of low dimensional ZnO nanostructures, including spheres, rods, sheets, and wires, were successfully synthesized using a simple and facile hydrothermal method assisted with different surfactants. Zinc acetate dihydrate was chosen as the precursors of ZnO nanostructures. We found that polyethylene glycol (PEG), polyvinylpyrrolidone (PVP), glycine, and ethylene glycol (EG) play critical roles in the morphologies and microstructures of the synthesized nanostructures, and a series of possible growth processes were discussed in detail. Gas sensors were fabricated using screen-printing technology, and their sensing properties towards acetylene gas (C_2_H_2_), one of the most important arc discharge characteristic gases dissolved in oil-filled power equipments, were systematically measured. The ZnO nanowires based sensor exhibits excellent C_2_H_2_ sensing behaviors than those of ZnO nanosheets, nanorods, and nanospheres, indicating a feasible way to develop high-performance C_2_H_2_ gas sensor for practical application.

## 1. Introduction

Acetylene gas (C_2_H_2_) is one of the most important arc discharge characteristic gases dissolved in oil-filled power equipments, such as power transformer, circuit breaker, and high-voltage bushing. Online monitoring of dissolved C_2_H_2_ gas plays a quite significant role in condition assessment and fault diagnosis of these power equipments [1–4]. According to the comparison and analysis of the detection data value of C_2_H_2_ gas, we can timely and effectively obtain the running status of the equipments [5–8].

Recently, interest in C_2_H_2_ gas detection has been extremely simulated and great attention has been made to this field [9–15]. Many of sensing technologies, including metal oxide semiconductor (MOS) [10], infrared [11], Raman [12] or photoacoustic [13, 14] spectroscopy, and carbon nanotube [15] have been employed for C_2_H_2_ detection. Owing to the remarkable advantages of simple fabrication process, rapid response and recovery time, low maintenance cost, and long service life, metal oxide semiconductors such as ZnO [16], SnO_2_ [17, 18], WO_3_ [19], TiO_2_ [20] have been proved to be promising sensing materials and widely used for gas detection [21, 40].

However, there still exist some limitations needed to be further improved, such as high operating temperature and low C_2_H_2_ sensing response, due to an extremely low C_2_H_2_ concentration (less than or equal to 5 *μ*L/L) dissolved in transformer, breaker, and bushing oil [22, 23]. Therefore, developing high-performance C_2_H_2_ gas sensor with high accuracy and low detection limit is currently the subject of intensive research. In recent years, low dimensional ZnO nanostructures, such as nanospheres [24], nanorods [25], nanoflowers [26], nanobelts [27], nanowires [28], nanotubes [29], nanoflakes [30]. and nanosheets [31], have been successfully prepared and widely reported. When compared to traditional bulk ZnO material, low dimensional ZnO nanostructures would show much more excellent catalytic [32, 33] and sensing performance [34, 35] due to high specific surface area, special hole, and pore structure. For instance, Chen and coworkers [30] synthesized the unique porous ZnO polygonal nanoflakes using microwave hydrothermal method and reported their improving NO_2_ gas sensing properties. Jang et al. [36] prepared low dimensional ZnO nanoplates with a high population of polar Zn (0001) faces through a soft-solution process, which demonstrated excellent photocatalytic activity for H_2_O_2_ generation.

To the best of our knowledge, reports on the synthesis of variety low dimensional ZnO nanostructures using hydrothermal method only by changing the active agent have been rare, and a systematically comparative study on their sensing properties towards C_2_H_2_ gas is a vacancy. Thus, in this study different morphologies of low dimensional ZnO nanostructures, including spheres, rods, sheets, and wires, are successfully synthesized via hydrothermal method assisted with various surfactants. X-ray powder diffraction (XRD) and field emission scanning electron microscopy (FESEM) were employed to characterize the prepared samples. A possible growth mechanism was discussed in detail. Finally, C_2_H_2_ gas sensing properties were systematically measured and its sensing mechanism was proposed.

## 2. Experimental

### 2.1. Preparation and Characterization of ZnO Nanostructures

Four morphologies of low dimensional ZnO nanostructures, including spheres, rods, sheets, and, wires were synthesized by the hydrothermal method assisted with different surfactants. All the raw chemicals were analytical-grade reagents purchased from Chongqing Chuandong Chemical Reagent Co., Ltd. and were directly used as received without any further purification. The detailed synthesis processes were represented as follows.

In a typical hydrothermal synthesis process of ZnO nanospheres, 1.0 mmol zinc acetate dihydrate (Zn(CH_3_COOH)_2_·2H_2_O), 2 mmol ammonium carbonate ((NH_4_)_2_CO_3_), and 0.1 g of citric acid were first mixed together and completely dissolved into 40 mL mixture of absolute ethanol and distilled water (1/1, V/V) in a 100 mL capacity beaker, followed by mild magnetic stirring for 30 min. Then the mixed solution was translated into a 100 mL of Teflon-lined stainless steel autoclave, sealed and heated at 140°C for 10 h in an electric furnace. When the hydrothermal reaction was completed, the autoclave was cooled to room temperature naturally. The prepared products were harvested by centrifugation, washing away the unwanted ions with distilled water and ethanol four times, respectively, and finally drying at 80°C in air for 12 h.

ZnO nanorods, nanosheets, and nanowires were obtained in a similar synthesis process of the as-synthesized ZnO nanospheres, except different surfactants which were employed to replace citric acid. In our study, 0.2 g of polyvinylpyrrolidone (PVP, K30) and 10 mL of polyethylene glycol (PEG, MW = 6000) were, respectively, added to the precursors to prepare ZnO nanorods and nanowires. Glycine (0.1 g) and 5 mL of EG were employed to prepare ZnO nanosheets.

X-ray powder diffraction (XRD) patters were performed on a Rigaku D/Max-1200X diffractometry (Tokyo, Japan) with Cu K*α* radiation (40 kV, 200 mA and *λ* = 1.5418 Å), and a scanning rate 0.02° s^−1^ from 20° to 80°. The morphologies and microstructures of the as-prepared nanostructures were characterized with a Nova 400 Nano field emission scanning electron microscope (FE-SEM, FEI, Hillsboro, OR, USA). The specific surface area and pore size of the prepared nanostructures were conducted with a surface area and porosimetry analyzer (V-Sorb 2800, Beijing Jinaipu General Instrument Co., Ltd., Beijing, China).

### 2.2. Fabrication and Measurement of Sensors

**Figure 1:**
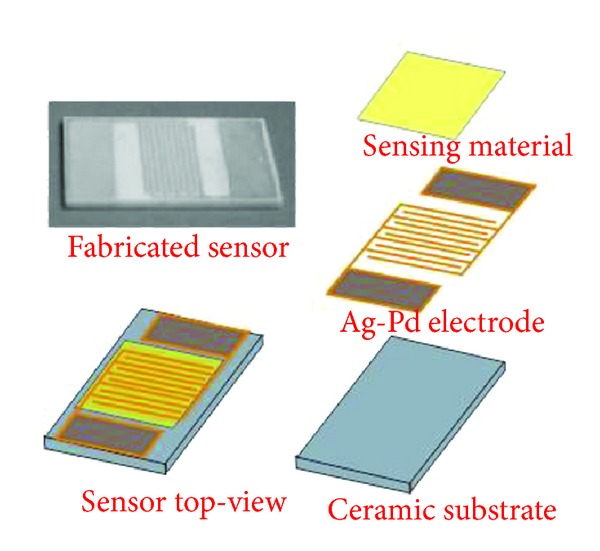
Structure representation of the fabricated gas sensor.

To investigate the sensing performances of the as-prepared ZnO nanostructures, gas sensors were fabricated with screen-printing technique [24, 34]. ZnO nanostructures were, respectively, further ground into fine powder and mixed with distilled water in a weight ratio of 8 : 2 to form a paste. The paste was subsequently screen-printed onto a planar ceramic substrate to form a sensing film and its thickness is about 50 *μ*m.  Figure 1 displays the schematic representation of the planar ceramic substrate, which was purchased from Beijing Elite Tech Co., Ltd., (Beijing, China). As shown in Figure 1 five pairs of Ag-Pd interdigital electrodes were preplaced on the planar ceramic substrate with a width of 0.2 mm. The length, width, and thickness of the planar ceramic substrate were 13.4, 7, and 1 mm, respectively [34]. The prepared gas sensing film was then dried in air to remove the unwanted impurities. Meanwhile, a 0.1 g of an ethyl cellulose solution in ethylester acetate was coated on the surface of the sensitive film as a protective layer to improve the sensor antipollution. Finally, the sensor was obtained after aging at 60°C for 48 h.

**Figure 2:**
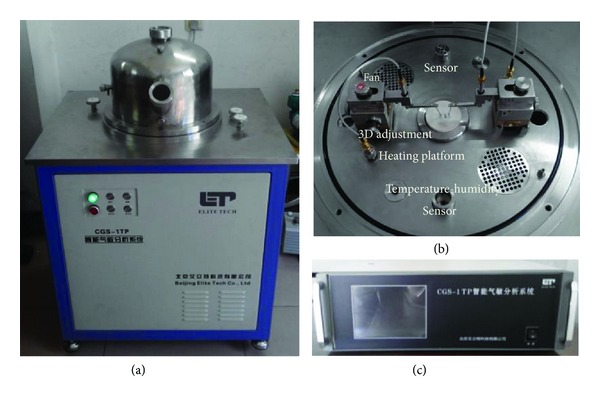
(a) The CGS-1TP gas sensing analysis system. (b) A photograph of the operating platform. (c) The control chamber of CGS-1TP.

The gas sensing properties of the sensors towards C_2_H_2_ gas were performed on a CGS-1TP (Chemical Gas Sensor-1 Temperature Pressure) intelligent gas sensing analysis system (Beijing Elite Tech Co., Ltd.) [34, 37, 38]. This multifunctional system shown in Figure 2 was mainly composed of the heating system, circulating cooling system, vacuum system, probe adjustment system, gas distribution system, measurement and data acquisition system, and measurement control software. The heating system and circulating cooling system could offer an external temperature control from room temperature to 500°C with an adjustment precision of 1°C. Figure 2 shows a top-view photograph of the operating platform, where the planar sensor was laid on the temperature control and two adjustable probes were pressed on the sensor electrodes to collect electrical signals. All sensors were preheated at different operating temperatures for about 30 min, and when the resistance value was stable certain concentration of C_2_H_2_ gas was injected into the test chamber (18 L in volume) by a microinjector through a rubber plug. The targeted gas was mixed with air in the test chamber by two fans. After its resistance value reached a new constant value, the test chamber was opened to recover the sensor in air. The whole experimental process was performed in a super-clean room with the constant humidity (25% relative humidity) and temperature (20°C), recorded automatically by the analysis system. The sensor resistance and sensitivity were also collected and analyzed by the system in real time.

The response value (*S*) of the sensor was designated as *S* = *R*_*a*_/*R*_*g*_ [37], where *R*_*a*_ was the resistance value of prepared sensor in air (base resistance) and *R*_*g*_ was that in a mixture of C_2_H_2_ gas and air. The time taken by the sensor to reach 90% of the total resistance change was defined as the response time when the target gas was introduced to the sensor or the recovery time when the ambience was replaced by air [38]. All measurements were repeated several times to ensure the repeatability and stability of the sensor.

## 3. Results and Discussion

### 3.1. Structural Characterization

To determine the crystalline phase and chemical composition of the prepared products, we first conducted the X-ray powder diffraction (XRD) analyses. Figure 2 shows the typical XRD patterns of the synthesized ZnO nanospheres, nanorods, nanosheets, and nanowires. It can be clearly seen from Figure 2 that the prominent peaks of (100), (002), (101), (102), and (110), and other smaller diffraction peaks, well correspond to the standard spectrum of wurtzite hexagonal ZnO structure (JCPDS card no. 36-1451, *a* = *b* = 3.249 Å and *c* = 5.206 Å). No diffraction peaks from any impurities were observed, revealing a high purity of the ZnO nanostructures under current synthetic conditions.

**Figure 3:**
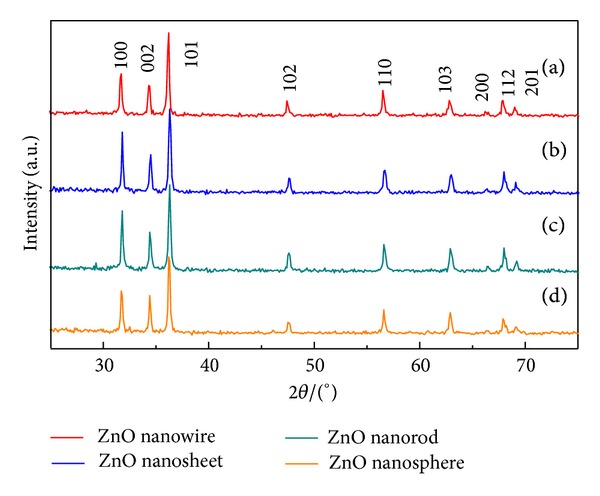
XRD diffraction patterns of the prepared ZnO nanostructures.

The morphologies and microstructures of the prepared samples were further investigated by field emission scanning electron microscopy (FESEM). Figures 3(a)–3(d) demonstrate the typical FESEM images of the synthesized ZnO nanospheres, nanorods, nanosheets, and nanowires, respectively.  Figure 3(a) shows an overview FESEM image of the sphere-like ZnO nanostructures, where one can clearly see that the ZnO nanospheres are highly uniform in size and shape with an average diameter of 400 to 500 nm.  Figure 3(b) displays a FESEM image of closely packed ZnO nanorods in rectangular geometrical shapes. The lengths and diameters of ZnO nanorods are calculated to be about 500~800 nm and 200~300 nm, respectively.  Figure 3(c) shows a typical FESEM image of ZnO nanosheets, which are nearly in irregular polygon shapes. It can be obviously seen in Figure 3(c) that the surface of nanosheets was rather smooth, and many nanosheets were distributed layer by layer in random orientations. The average thickness of these chaotic nanosheets was measured to be about 120~150 nm.  Figure 3(d) reveals the as-prepared ZnO nanowires, where a wide range of ZnO nanowires were joined together and formed a net-like structure. The diameter of individual wire ranges from 200 to 350 nm, and its length ranges from hundreds of nanometers to tens of micrometers. The average diameter of these nanowires is around 250 nm. These results demonstrate that variety morphologies of low dimensional ZnO nanostructures has been successfully prepared via a simple hydrothermal method with different surfactants.

### 3.2. Growth Mechanism

Surfactant plays an important role in the final morphology of our products. In order to reveal how the surfactants affect the morphologies of the synthesized ZnO nanostructures, a series of possible growth processes were discussed as follows. Zn(OH)_4_^2−^ have been reported as a precursor for hydrothermal synthesis of ZnO nanostructures from aqueous solution [21, 39]. The chemical reactions of forming Zn(OH)_4_^2−^ and ZnO nanostructures in the reaction system could be represented by the following chemical reactions [40]:

(1) $$\begin{array}{c} {\text{Zn}}^{2+}+4{\text{OH}}^{-}⟶\text{Zn}{{\left(\text{OH}\right)}_{4}}^{2-}\\ \text{Zn}{{\left(\text{OH}\right)}_{4}}^{2-}⟶\text{ZnO}+{\text{H}}_{2}\text{O}+2{\text{OH}}^{-}. \end{array}$$

**Figure 4:**
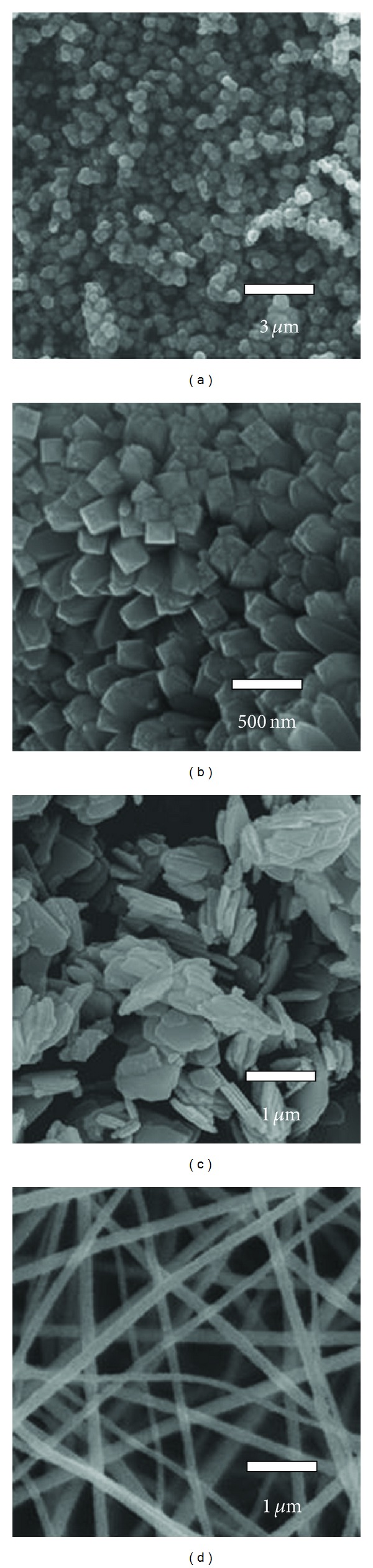
FESEM images of the prepared ZnO nanostructures: (a) sphere, (b) rod, (c) sheet, and (d) wire.

The growth process can be divided into the nucleation stage and the self-assembly stage, respectively, closely related to the reaction solvents and the surface-active agents. At first numerous Zn(CH_3_COOH)_2_ and (NH_4_)_2_CO_3_ were hydrolyzed in the reaction solution, and abundant component Zn(OH)_4_^2−^ ions appeared. And a large number of tiny ZnO nanoparticles spontaneously formed from the dehydration of Zn(OH)_4_^2−^ ions. With the reaction time increasing, many tiny ZnO crystal nanoparticles gathered together and developed into larger crystals. Thus, without any surfactant tiny ZnO particles assembled into spherical ZnO nanostructures as shown in Figure 4(a). As we know PVP is a nonionic surfactant, and an easily polarized functional group “–C=O” is universally present in its repeated unit [34]. The “O” atom in the “–C=O” functional group of PVP has a negative charge and the “Zn” in the precursor particles is positively charged, so an intense attraction occurs between “O” atom of PVP and “Zn” atom of ZnO, which played a critical role in controlling the morphology of ZnO nanorods [41]. With the reaction time increasing, a closely packed ZnO nanorod appeared.

When certain amount of glycine and EG were added to the reaction solvent, ZnO nanosheets were obtained as seen in Figure 4(c). It could be considered that glycine agglomerated numerous tiny ZnO nanoparticles to form smooth plates in horizontal direction, and EG acted as a template which limited the growth of tiny ZnO nanoparticles along vertical direction. Therefore, ZnO nanosheets were synthesized by current hydrothermal method assisted with surfactant glycine and EG assisted. As a kind of low level organic polymer with liquid state, PEG is usually used as a significant surface-active agent to synthesis nanomaterials. When PEG was added to the precursor reaction solvent, the net-like ZnO nanowires were obtained as displayed in Figure 4(d). As we know PEG has a long-chain structure and numerous hydrophilic “–O–” and “–CH_2_–CH_2_–” groups exist along its long chains [28, 42]. In the reaction process, tiny Zn(OH)_4_^2−^ and ZnO nanoparticles embedded into PEG long-chain substrate and easily grew along the long chain of PEG. So under this hydrothermal treatment, individual ZnO nanowire was synthesized along the PEG long chain and a great amount of nanowires clustered chaotically into a novel three-dimensional net-like ZnO nanowires.

### 3.3. Gas Sensing Properties

Gas sensors were fabricated by screen-printing technique based on the prepared ZnO nanospheres, nanorods, nanosheets, and nanowires, respectively [25]. C_2_H_2_ sensing properties of these fabricated sensors were systematically measured to gain insight into how the various morphologies of ZnO impact C_2_H_2_ sensing properties.

**Figure 5:**
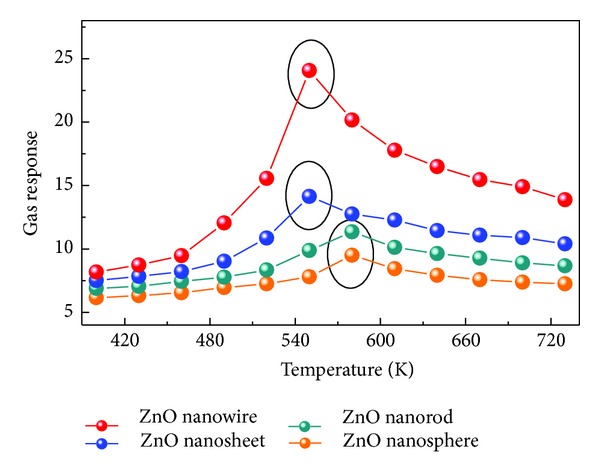
Gas response of the sensors to 20 *μ*L/L of C_2_H_2_ gas as a function of operating temperature ranging from 400 K to 730 K.

Figure 5 presents the gas responses of the prepared sensors to 20 *μ*L/L of C_2_H_2_ gas as a function of operating temperature ranging from 400 to 730 K. For each response curve of the four sensors, a similar changing trend was tested. The C_2_H_2_ response curve increases first in various degrees and reaches its maximum value and then decreases rapidly with further increasing temperature. As shown in Figure 5, the optimum operating temperatures of the prepared ZnO nanowires and nanosheets were tested to be about 550 K, while it was suggested to be about 580 K for ZnO nanospheres and nanorods, attributed to the sensor which exhibits the maximum response at this temperature. And the corresponding C_2_H_2_ responses are 24.07 and 14.14 for ZnO nanowires and nanosheets, much higher than nanospheres (11.34) and nanorods (9.53). These results imply that the synthesized ZnO nanowires and nanosheets demonstrate not only lower operating temperature but also higher response than nanospheres and nanorods.

**Figure 6:**
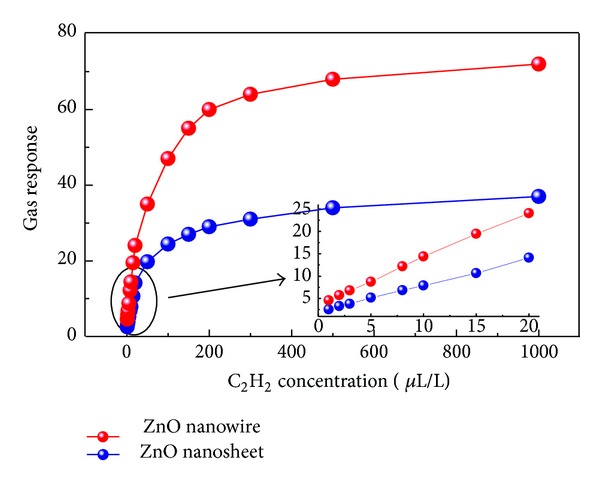
Gas response of the sensors to different C_2_H_2_ gas concentrations (1–1000 *μ*L/L) at 550 K; the inset shows the corresponding sensor response curve (1–20 *μ*L/L).

To further understand the difference of ZnO nanowires and nanosheets sensing properties, ZnO nanowires and nanosheets based sensors were exposed to different concentrations of C_2_H_2_ gas at 550 K. As seen in Figure 6 for both sensors the response curve increases rapidly in linearity with C_2_H_2_ concentration below 50 *μ*L/L, exhibits a quasilinearity from 50 to 200 *μ*L/L, and finally reaches saturation at nearly about 1000 *μ*L/L. Although similar C_2_H_2_ response curve was displayed in Figure 6 for ZnO nanosheets, while much weaker than that of nanowires, the insert one shows C_2_H_2_ sensing response in low concentration from 1 to 20 *μ*L/L. Such a high linear dependence implies that our prepared sensors can be employed to onsite detect C_2_H_2_ gas in oil-filled power equipments.

**Figure 7:**
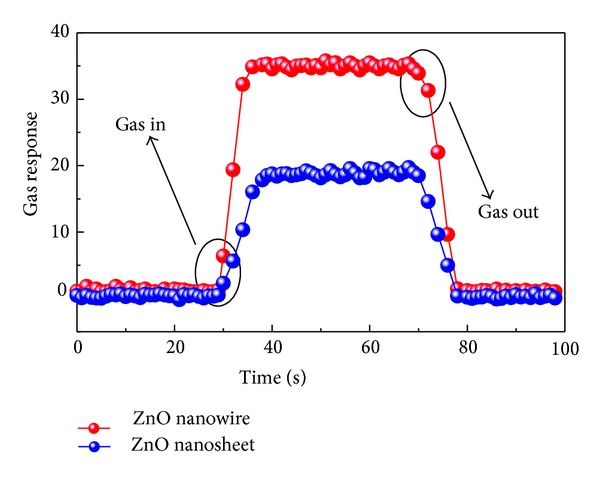
Response transients of the sensors to 20 *μ*L/L of C_2_H_2_ at 550 K.

Figure 7 presents a typical response-recovery curve of ZnO nanowires and nanosheets based sensors exposed to 20 *μ*L/L of C_2_H_2_ atmosphere optimized at 550 K. As seen in Figure 7 the response increases sharply when gas was injected in (gas in) and returns to its original state when the test chamber was opened to recover (gas out). In light of the response-recovery time defined in Section 2.2, the response-recovery time of ZnO nanowires and nanosheets was calculated to be about 6–12 and 12–18, respectively. Such rapid response-recovery property meets the basic demands for the engineering application of C_2_H_2_ detection.

**Figure 8:**
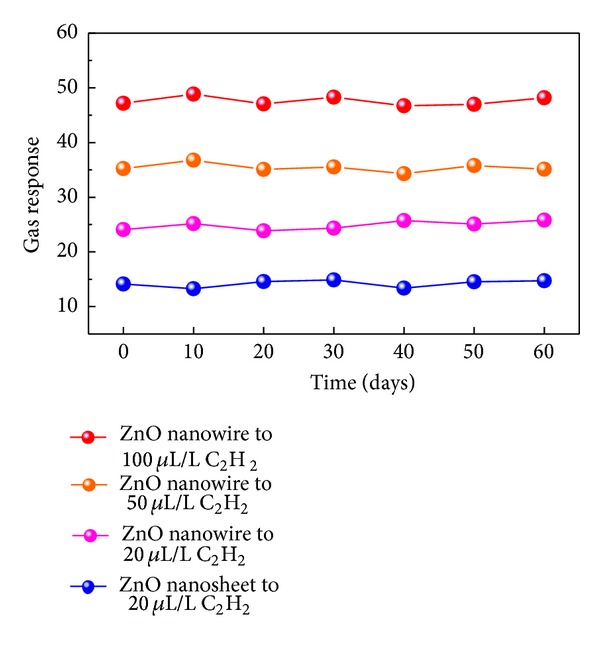
Stability and repeatability of the sensors to 20, 50, and 100 *μ*L/L of C_2_H_2_ at 550 K.

The sensing measurements of ZnO nanowires to 20, 50, and 100 *μ*L/L of C_2_H_2_ and ZnO nanosheets to 20 *μ*L/L of C_2_H_2_ were repeated every 10 days in two months to conduct the long-time stability. As shown in Figure 8 the ZnO nanowires and nanosheets exhibit nearly constant sensing behaviors to C_2_H_2_ gas during the long experimental cycles, confirming the excellent stability of our sensors.

**Figure 9:**
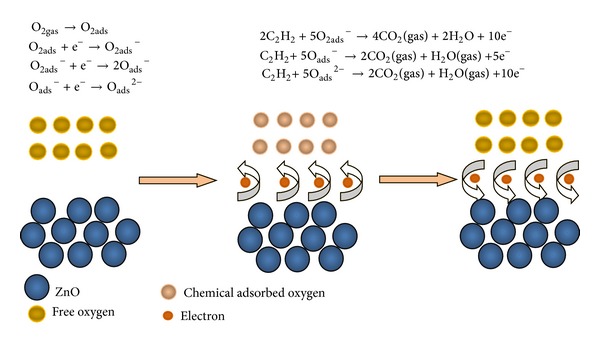
Schematic diagram of the gas-sensing reaction process.

C_2_H_2_ sensing process can be understood as follows and the detailed gas-sensing reaction process was represented in Figure 9. It is known to all that the ZnO is a typical n-type semiconducting material and its gas sensing properties are predominantly controlled by the change in surface resistance. Due to the nonstoichiometry of the prepared ZnO nanostructures, many oxygen vacancies extensively exist in their crystal lattices [21]. The oxygen vacancy in ZnO acts as an electron donor to provide electrons to conduction band. When the ZnO nanostructures were surrounded by air, free oxygen molecules would be absorbed on the sensing surface. Adsorption oxygen molecules would capture free electrons from ZnO conduction band to generate various kinds of chemical adsorbed oxygen species, including O_2_^−^, O^2−^, and O^−^ [40]. Consequently, depletion layers are formed in the surface area of ZnO nanostructures, decreasing the number of change carrier and electron mobility in ZnO conduction band, and resulting in a higher electrical resistance. When ZnO nanostructures were exposed to certain concentration of C_2_H_2_, an oxidation-reduction reaction happened between the chemical adsorbed oxygen and C_2_H_2_ gas. In this reaction the preadsorbed free electrons are released to ZnO conduction band, decreasing the height of barrier in the depletion region and increasing its carrier concentration in the conduction band. Thus, a decreased electrical resistance of the sample was measured.

According to gas sensing results of our products tested above, it can be concluded that low dimensional ZnO nanowires sensor exhibits excellent C_2_H_2_ sensing properties than those of nanowires, nanorods, and nanospheres. A nitrogen adsorption and desorption measurement was employed to test the texture properties. Based on the Barrett-Joyner-Halenda (BJH) method [24] the calculated BET surface area and pore structure parameters are demonstrated in Table 1. With such higher specific surface area and pore diameter, the net-like ZnO nanowires would offer much more adsorption positions for gas sensing reaction and easily enable gas molecule to adsorb on the surface [21, 40]. Simultaneously, the one-dimensional structure of nanowire can facilitate fast mass transfer of the free electrons along the one-dimensional wire direction [43, 44]. Therefore, the ZnO nanowires sensor shows lower optimum operating temperature, higher C_2_H_2_ sensing response together with rapid response-recovery characteristic, and good long-term stability than the other three ZnO nanostructures.

## 4. Conclusion

A simple and facile hydrothermal method was employed to synthesize various low dimensional ZnO nanospheres, nanorods, nanosheets, and nanowire only by changing the surfactants. A possible growth mechanism was discussed and we found that PEG, PVP, glycine, and EG play significant roles in the morphology control of ZnO nanowires, nanorods, and nanosheets, respectively. Gas sensors were fabricated with screen-printing technology and C_2_H_2_ sensing properties were systematically measured. The nanowires and nanosheets sensors demonstrate a lower optimum operating temperature of 550 K than nanospheres and nanorods. And the corresponding response values (20 *μ*L/L C_2_H_2_ gas) are measured to be about 24.07, 14.14, 11.34, and 9.53, respectively. Moreover, the ZnO nanowires sensor exhibits a high linear dependence in the measurement scope and its detection limit is low up to 1 *μ*L/L. These results indicate that the prepared nanowires exhibit excellent gas sensing properties than those of nanosheets, nanorods, and nanospheres, providing us a feasible way to develop high-performance C_2_H_2_ gas sensor by tailoring the surface morphology and structure of the nanostructures.

## Figures and Tables

**Table 1:** BET surface area and pore structure parameters of the samples.

ZnO nanostructures	*S*_ BET_ (m^2^g^−1^)	*v*_*P*_ (cm^2^g^−1^)	*d*_*P*_ (nm)
Wire	29.4	0.06	16.8
Sheet	25.3	0.04	12.6
Rod	21.7	0.02	9.7
Sphere	18.8	0.02	8.5

## References

[1] Akbari A, Setayeshmehr A, Borsi H, Gockenbach E, Fofana I (2010). Intelligent agent-based system using dissolved gas analysis to detect incipient faults in power transformers. *IEEE Electrical Insulation Magazine*.

[2] Arakelian VG (2004). The long way to the automatic chromatographic analysis of gases dissolved in insulating oil. *IEEE Electrical Insulation Magazine*.

[3] Duval M, Dukarm JJ (2005). Improving the reliability of transformer gas-in-oil diagnosis. *IEEE Electrical Insulation Magazine*.

[4] Singh S, Bandyopadhyay M (2010). Dissolved gas analysis technique for incipient fault diagnosis in power transformers: a bibliographic survey. *IEEE Electrical Insulation Magazine*.

[5] Chen W, Pan C, Yun Y, Liu Y (2009). Wavelet networks in power transformers diagnosis using dissolved gas analysis. *IEEE Transactions on Power Delivery*.

[6] Khan I-U, Wang Z, Cotton I, Northcote S (2007). Dissolved gas analysis of alternative fluids for power transformers. *IEEE Electrical Insulation Magazine*.

[7] Morais DR, Rolim JG (2006). A hybrid tool for detection of incipient faults in transformers based on the dissolved gas analysis of insulating oil. *IEEE Transactions on Power Delivery*.

[8] Duval M (2008). The duval triangle for load tap changers, non-mineral oils and low temperature faults in transformers. *IEEE Electrical Insulation Magazine*.

[9] Cheung A, Johnstone W, Moodie D (2006). Gas detection based on optical correlation spectroscopy using a single light source. *Measurement Science and Technology*.

[10] Tamaekong N, Liewhiran C, Wisitsoraat A, Phanichphant S (2011). Acetylene sensor based on Pt/ZnO thick films as prepared by flame spray pyrolysis. *Sensors and Actuators B*.

[11] Wu ZY, Yu QX (2013). Photoacoustic spectroscopy detection and extraction of discharge feature gases in transformer oil based on 1.5 *μ* tunable fiber laser. *Infrared Physics and Technology*.

[12] Li XY, Xia YX, Huang JM, Zhan L (2008). A Raman system for multi-gas-species analysis in power transformer. *Applied Physics B*.

[13] Yun Y, Chen W, Wang Y, Pan C (2008). Photoacoustic detection of dissolved gases in transformer oil. *European Transactions on Electrical Power*.

[14] Chen W-G, Liu B-J, Huang H-X (2011). Photoacoustic sensor signal transmission line model for gas detection in transformer oil. *Sensor Letters*.

[15] Zhang XX, Zhang JB, Li RH, Liao YF (2013). Application of hydroxylated single-walled carbon nanotubes for the detection of C_2_H_2_ gases in transformer oil. *Journal of Computational and Theoretical Nanoscience*.

[16] Zhang J, Wang S, Xu M (2009). Hierarchically porous ZnO architectures for gas sensor application. *Crystal Growth and Design*.

[17] Zeng W, Liu T, Liu D, Han E (2011). Hydrogen sensing and mechanism of M-doped SnO_2_(M = Cr^3+^, Cu^2+^andPd^2+^) nanocomposite. *Sensors and Actuators B*.

[18] Liu D, Liu T, Zhang H, Lv C, Zeng W, Zhang J (2012). Gas sensing mechanism and properties of Ce-doped SnO_2_ sensors for volatile organic compounds. *Materials Science in Semiconductor Processing*.

[19] You L, Sun YF, Ma J (2011). Highly sensitive NO_2_ sensor based on square-like tungsten oxide prepared with hydrothermal treatment. *Sensors and Actuators B*.

[20] Yu Y, Xu D (2007). Single-crystalline TiO_2_ nanorods: highly active and easily recycled photocatalysts. *Applied Catalysis B*.

[21] Huang J, Wan Q (2009). Gas sensors based on semiconducting metal oxide one-dimensional nanostructures. *Sensors*.

[22] Chen WG, Zhou Q, Gao TY, Su XP, Wan F (2013). Pd-doped SnO_2_-based sensor detecting characteristic fault hydrocarbon gases in transformer oil. *Journal of Nanomaterials*.

[23] Qi Q, Zhang T, Zheng X (2008). Electrical response of Sm_2_O_3_-doped SnO_2_ to C_2_H_2_ and effect of humidity interference. *Sensors and Actuators B*.

[24] Guo WW, Liu TM, Sun R, Chen Y, Zeng W, Wang ZC (2013). Hollow, porous, and yttrium functionalized ZnO nanospheres with enhanced gas-sensing performances. *Sensors and Actuators B*.

[25] Qi Q, Zhang T, Yu Q (2008). Properties of humidity sensing ZnO nanorods-base sensor fabricated by screen-printing. *Sensors and Actuators B*.

[26] Xing L-L, Ma C-H, Chen Z-H, Chen Y-J, Xue X-Y (2011). High gas sensing performance of one-step-synthesized Pd-ZnO nanoflowers due to surface reactions and modifications. *Nanotechnology*.

[27] Xing GZ, Fang XS, Zhang Z (2010). Ultrathin single-crystal ZnO nanobelts: ag-catalyzed growth and field emission property. *Nanotechnology*.

[28] Ko SH, Lee D, Kang HW (2011). Nanoforest of hydrothermally grown hierarchical ZnO nanowires for a high efficiency dye-sensitized solar cell. *Nano Letters*.

[29] Wang JX, Sun XW, Yang Y, Wu CML (2009). N-P transition sensing behaviors of ZnO nanotubes exposed to NO_2_ gas. *Nanotechnology*.

[30] Chen M, Wang Z, Han D, Gu F, Guo G (2011). Porous ZnO polygonal nanoflakes: synthesis, use in high-sensitivity NO_2_ gas sensor, and proposed mechanism of gas sensing. *The Journal of Physical Chemistry C*.

[31] Zeng Y, Qiao L, Bing YF (2012). Development of microstructure CO sensor based on hierarchically porous ZnO nanosheet thin films. *Sensors and Actuators B*.

[32] Leschkies KS, Divakar R, Basu J (2007). Photosensitization of ZnO nanowires with CdSe quantum dots for photovoltaic devices. *Nano Letters*.

[33] Lin D, Wu H, Zhang R, Pan W (2009). Enhanced photocatalysis of electrospun Ag-ZnO heterostructured nanofibers. *Chemistry of Materials*.

[34] Zhou Q, Chen WG, Xu LN, Peng SD (2013). Hydrothermal synthesis of various hierarchical Zno nanostructures and their methane sensing properties. *Sensors*.

[35] Jing Z, Zhan J (2008). Fabrication and gas-sensing properties of porous ZnO nanoplates. *Advanced Materials*.

[36] Jang ES, Won J-H, Hwang S-J, Choy J-H (2006). Fine tuning of the face orientation of ZnO crystals to optimize their photocatalytic activity. *Advanced Materials*.

[37] Zhang X-J, Qiao G-J (2012). High performance ethanol sensing films fabricated from ZnO and In_2_O_3_ nanofibers with a double-layer structure. *Applied Surface Science*.

[38] Feng CH, Li W, Li C, Zhu LH, Zhang HF (2012). Highly efficient rapid ethanol sensing based on In_2−x_N_x_O_3_ nanofibers. *Sensors and Actuators B*.

[39] Hu H, Huang X, Deng C, Chen X, Qian Y (2007). Hydrothermal synthesis of ZnO nanowires and nanobelts on a large scale. *Materials Chemistry and Physics*.

[40] Arafat MM, Dinan B, Akbar SA, Haseeb ASMA (2012). Gas sensors based on one dimensional nanostructured metal-oxides: a review. *Sensors*.

[41] Zeng M, Yin H, Yu K (2012). Synthesis of V_2_O_5_ nanostructures with various morphologies and their electrochemical and field-emission properties. *Chemical Engineering Journal*.

[42] Yin Y-X, Jiang L-Y, Wan L-J, Li C-J, Guo Y-G (2011). Polyethylene glycol-directed SnO_2_ nanowires for enhanced gas-sensing properties. *Nanoscale*.

[43] Qi Q, Zhang T, Liu L, Zheng X (2009). Synthesis and toluene sensing properties of SnO_2_ nanofibers. *Sensors and Actuators B*.

[44] Chen WG, Zhou Q, Xu LN, Wan F, Peng SD, Zeng W (2013). Improved methane sensing properties of Co-doped SnO_2_ electrospun nanofibers. *Journal of Nanomaterials*.

